# Undernutrition and its associated factors among children aged 6 to 59 months in Menz Gera Midir district, Northeast Ethiopia: A community-based cross-sectional study

**DOI:** 10.1371/journal.pone.0278756

**Published:** 2022-12-06

**Authors:** Getabalew Engidaye, Melak Aynalem, Tiruneh Adane, Yemataw Gelaw, Aregawi Yalew, Bamlaku Enawgaw

**Affiliations:** School of Biomedical and Laboratory Sciences, Department of Hematology and Immunohematology, College of Medicine and Health Science, University of Gondar, Gondar, Ethiopia; James Cook University, AUSTRALIA

## Abstract

**Background:**

Undernutrition can lead to impaired physical growth, restricted intellectual skills, low school performance, reduced working capacity, and rooted disability in adult life. Thus, this study was designed to assess the prevalence and associated factors of undernutrition among children aged 6 to 59 months.

**Methods:**

A community-based cross-sectional study was conducted among 432 children aged 6 to 59 months in the Menz Gera Midir District. A multi-stage sampling technique was applied to recruit the study participants. Socio-demographic and socio-economic variables were collected by using structured questionnaires. Anthropometric measurements of the children were measured according to the World Health Organization’s recommendation. A data collection sheet was used to collect information on the types of foods and number of meals consumed by the child. A bivariable and multivariable logistic regression was performed to identify factors associated with undernutrition.

**Result:**

In this study, about 11.3% (95% CI: 8.3–14.3%), 50.2% (95% CI: 45.5–55.0%), and 28% (95% CI: 23.8–32.3%) were wasted, stunted, and underweight, respectively. Children aged 12–23 months (AOR: 1.97; 95% CI: 1.01–3.87), 36–47 months (AOR: 2.05; 95% CI: 1.00–4.19), and being anemic (AOR: 2.92; 95% CI: 1.73–4.92) were found to be an independent predictor of stunting. Moreover, being anemic was found to be significantly associated with wasting (AOR: 6.84; 95% CI: 3.16–14.82).

**Conclusion:**

According to the findings of this study, undernutrition was a serious public health issue among 6–59 month old children in the Menz Gera Midir District. Children’s age and anemia status were significantly associated with stunting and wasting. Therefore, community-based nutrition programs are vital to reduce childhood undernutrition

## Background

Malnutrition is a global public health issue that affects every country [1]. It is caused by unbalanced diets that lack all the necessary nutrients (macronutrients and micronutrients), a condition called undernutrition. It is also caused by excessive consumption of nutrients, which is called "over nutrition" [2,3].

Undernutrition can be classified as stunting, wasting, and being underweight. Stunting is characterized by low height-for-age (HAZ) and is the result of long-term nutritional deficiency. Wasting is low weight-for-height (WHZ), which indicates short-term poor nutritional status. On the other hand, underweight is a low weight-for-age (WAZ) that shows reduced public situations in both the long and short term [4]. Undernutrition has both short-term and long-term consequences. Of these consequences, child undernutrition could cause decreased physical growth, restricted intellectual skills, low academic performance, reduced working ability at maturity, lead to chronic illness and disability in adult life, and affect national economic growth [5] Also, there is an increased risk of cardiovascular disease, diabetes mellitus, cancer, and mental health issues in adulthood [6].

Globally, about 8–11 million preschool children die each year, and more than 35% of these deaths are due to undernutrition [4]. According to the 2020 World Health Organization (WHO) report, about 149 million children under 5 were stunted, 45 million were wasted, and 38.9 million were overweight globally [7]. Undernutrition contributes to more than 50% of the child mortality rate in developing nations [5]. Furthermore, Asia and Africa were the most heavily impacted by wasting [8]. The levels of wasting and stunting in preschool children have become the highest problem in developing countries like Ethiopia [9]. Data from the Ethiopia demographic and health survey (EDHS) in 2019 showed that the prevalence of stunting, wasting, and underweight in preschool children was 38%, 10%, and 24%, respectively [10].

Studies on the associated factors of undernutrition showed that being male, parents with lower educational level, families with poor socioeconomic status, paternal occupation type, living in a large family size, having more than two offspring, children having non-breast milk feeding practice, children who had diarrhea, children whose mother had never visited an antenatal clinic, children who received butter as pre-lacteal feeding, the family did not follow family planning, and using an unprotected water source for drinking were some of the predictors of undernutrition [11–20].

Undernutrition is still a high-magnitude problem in developing countries. However, there is limited data regarding the nutritional status of preschool children in the Menz Gera Midir District. Therefore, this study was aimed at determining the prevalence and associated factors of undernutrition among preschool children in Menz Gera Midir District. As a result, researchers specializing in child health and nutrition, health policymakers, and other undernutrition experts will benefit from this study.

## Materials and methods

### Study setting, design and period

A community-based cross-sectional study was done in the Menz Gera Midir district from January to May 2017. The Menz Gera Midir district is located in the North Shewa zone of the Eastern Amhara Region of Ethiopia. The administrative center of the district is Mehal Meda town. The town has an elevation of 3037 meters above sea level with a latitude and longitude of 10018172 N/390 312. Based on the central statistics agency of Ethiopia, the district has a total population of 138,708 people, of whom 67,567 are men and 16,361 are urban inhabitants [21]. Approximately 46,235 households are found within the 28 smallest administrative units of the district. Of these, 5453 are urban households. In 2020, there were 13,422 children under the age of five [22].

### Study population

All children aged 6–59 months residing in Menz Gera Midir District were taken as a source population, whereas children aged 6–59 months in Menz Gera Midir District who volunteered to participate during the study period were considered as a study population. Children aged 6–59 months residing in the selected smallest administration unit for at least 6 months and whose parents/guardians were willing to fully participate in the study were included in this study. On the other hand, children with mental illnesses and severely ill children who could not give a response were excluded from the study.

### Study variables

In this study, wasting, stunting, and underweight were considered as dependent variables. While socio-demographic characteristics of preschool children (age group, sex) and respective parents/caregivers such as the mother’s sex, education, religion, marital status, occupation, family size, place of residence, socioeconomic status of the households, presence of anemia, intestinal infection, access to clean water, use of a toilet, vitamin A supplementation, vaccination status, and duration of breastfeeding practice of the mother were taken as independent variables.

### Sample size determination and sampling technique

A single population proportion formula was used to decide the sample size, and the expected prevalence of undernutrition was set at 25%, taken from Menz Keya [23], and the marginal error (d) of 5% and a 95% confidence interval were used to calculate the sample size. Furthermore, considering the affordable resources for the investigations, a design effect of 1.5 was applied, and the final sample size became 432. A multi-stage sampling technique was used to assess the prevalence of undernutrition in children aged 6–59 months. First, from all the smallest administrative units, about 25% were selected, which is seven, and then, from seven, one from urban and six from rural areas were recruited by a simple random sampling technique. In the second stage, a proportional sample allocation was applied to seven of the smallest administrative units, and a systematic sampling technique was used to select the total study participants. During the systematic sampling technique, the first study participant was selected by the lottery method, and the next study participant was selected by using the Kth interval. If there were two or more eligible children in the household, we used only one child by using the lottery method. If the selected household is closed, we returned to the household for a second visit, and if it is closed again, we moved on to the next household.

### Operational definitions

- Anemia: When hemoglobin (Hgb) level is less than 11g/dl for both sex [24,25]
- Mildly undernutrition: when weight for height/length is between -1SD and 1SD
- Moderately undernutrition: weight for height/length is between -2SD and -3SD with no edema
- Severely undernutrition: the presence of edema of both feet, or severe wasting or both (weight for height/length ≤ -3SD)
- Anthropometric indices: these are calculated from anthropometric measurements of weight, height and age
- Stunting: When young children have a low HAZ <-2SD
- Underweight: When children are too light for their age (WAZ) <-2SD
- Wasting: When children have a low WHZ <-2SD [24,25].

### Data collection procedures and laboratory methods

#### Household demographic and socio-economic data collection

The socio-demographic data of children (age, sex, residence, delivery status, birth order, and vaccination status) and socio-demographic and socio-economic data of the parents or caretakers of the child (age, sex, household wealth, occupation, religion, and marital status) were collected by a questionnaire which was prepared from the national survey and accordingly modified based on the reviewed literature [26]. In addition, food and nutritional technical assistance, the Helen Keller international food frequency questionnaire, and a 24-hour dietary recall questionnaire were used to assess household food security, food consumption pattern, and dietary diversity scores [27–29]. The questionnaire has five sections which contain socio-demographic, breastfeeding practices, household wealth index related questions, food and dietary frequency related questions, and questions on morbidity and vaccination data of the child. To complete this questionnaire, about 15 to 20 minutes are required.

#### Food consumption patterns

A qualitative household food security and dietary diversity score questionnaire was used to collect information on the types of foods and number of meals consumed by the index child over the past 24 hours. Probing questions were used to get information on the food types consumed, ingredients used to prepare meals, the type of snacks used, and the number of times the child ate the particular food within 24 hours of recall [30].

#### Anthropometric data for nutritional status

Anthropometric measurements such as children’s weight and height, as well as their mid-upper arm circumference (MUAC), were taken in accordance with a WHO recommendation from 2006 [31]. Children’s weight was measured through light-weight wear. The weight of children aged 24–59 months was measured to the nearest 0.1 kg by beam balance. For children aged less than 24 months, weight was measured to the nearest 0.1 kg using the Salter scale. A measuring board was used to measure the length and height; the height of children aged 6–23 months was measured in a recumbent position to the nearest 0.1 cm, while the height of children aged 24–59 months was measured to the nearest 0.1 cm while standing straight on a horizontal surface with their heels together and eyes straight forward. Anthropometric data from children was entered into the WHO Anthro software version 3.2.2.1. The WHO multi-center growth reference standards for the Z-scores of indices, such as WAZ, WHZ, and HAZ, were calculated. The children were classified as stunted, underweight, and wasted when their HAZ, WAZ, and WHZ scores were less than 2 SD from the median of the reference population, respectively [24].

#### Sample collection and laboratory investigation

*Measurement of Hgb*. Blood samples from capillary blood were sampled from all preschool children and the Hgb value was determined by a Hemocue analyzer (Hb 301+, Norway), which is recommended by WHO to measure population anemia prevalence [32]. The study area was located > 1000 m above sea level, and the results of Hgb were adjusted to its respective sea level (altitude) as recommended by WHO [25].

*Stool examinations*. Stool samples were collected from each study participant. Stool samples were labeled, clean, leak-proof containers. The wet mount was done with normal saline and direct microscopy was observed by using a light microscope. A formal ether concentration technique was performed for further parasitic detection.

#### Data management and quality control

The questionnaire, which was originally developed in English, was translated into the Amharic language and back-translated into English to ensure its consistency. Then the questioner was pretested by Dija’s smallest administration unit. Training was given for the data collectors on data recording, handling, and managing. The completeness and legibility of every questionnaire were checked daily by the principal investigator.

The weight measurement scales were adjusted daily (using standardized weighing grams) before use. All the measurements of weight and height were taken twice, and the average weight was taken. The cleanliness of microscope lenses (eyepiece and objective) was maintained by performing daily cleaning. Anthropometric measurements were taken twice, and the average of the two was taken.

Appropriate labeling, storage, packaging, and transportation methods and daily internal quality control with the known results that were available in the laboratory were implemented before use in and out of the laboratory. Recommended containers and collection procedures were employed. For delayed samples, an appropriate preservative (10% formalin for stool) was used for stability. A sample of inappropriate preservatives, transportation media, and temperature, and inadequate volume was rejected.

#### Data processing and analysis

Firstly, data was entered and stored using EPI-info version 7 and checked for consistency. The data was sorted and cleaned before being exported to the Statistical Package for Social Sciences (SPSS) version 25 for further analysis. Also, WHO Anthro software version 3.2.2.1 was used for anthropometric indices. Descriptive statistics such as frequencies, means, medians, and standard deviations were used to summarize the characteristics of the study participants. To identify factors associated with undernutrition a bivariable and multivariable logistic regression was performed. The bivariable logistic regression analyses were done to determine factors associated with wasting, stunting, and underweight. Predictor variables having a p-value less than or equal to 0.2 in the bivariable analysis were included in the multivariable analysis to control the confounders. A Hosmer-Lemeshow goodness-of-fit test was used to assess the model’s fitness. The crude odds ratio (COR) and adjusted odds ratio (AOR), along with the 95% confidence interval (CI), were used to determine the strength of association between the predictors and dependent variables. Variables with a p-value of less than 0.05 were considered statistically significant.

### Ethical considerations

Ethical clearance and approval was granted by the University of Gondar, College of Medicine and Health Sciences, School of Biomedical and Laboratory Sciences Ethical Review Committee (Reference number SBMLS/625/09). A permission letter was also obtained from the district health office and each village administrative office. The purpose of the research was explained to the study subjects and written informed consent was obtained from the mother or guardian of the child, and then those who were willing to participate were included in the study. Participation was fully voluntarily and refusal at any time during data collection was permitted. Information obtained in any course of study was kept confidential. Confidentiality was maintained by numeric coding of questionnaires. Any abnormal findings were linked to the nearby health center. Additionally, when the hemoglobin level of the children was below 11 g/dL, their mothers/care givers were informed to take them to a health facility for follow-up care.

## Results

### Child socio-demographic and health related characteristics

In this study, a total of 432 households, 390 (90.3%) from rural areas and 42 (9.7%) from urban areas, were included. Of them, 227 (52.5%) and 27 (6.3%) were females from rural and urban residences, respectively. The median age of the children was 24, IQR (14–42) months, with a median of the birth interval from their preceding elders, IQR (14–42). Moreover, 41 (9.5%) and 210 (48.6%) of the children were positive and negative for parasite infection, respectively, whereas 181 (41.9%) of the children were not assessed for parasitic infection. Concerning the anemia status of the children, 123 (28.5%) of the children were anemic (Table 1).

**Table 1:** Socio-demographic and health related characteristics of children aged 6 to 59 months in the Menz Gera Midir district Northeast Ethiopia in 2021, (N = 432).

Variables	Category	Frequency (n)	Percentage (%)
**Sex**	Male	178	41.2
	Female	254	58.8
**Age (months)**	6–11	90	16.7
	12–23	107	25.0
	24–35	70	17.1
	36–47	76	18.8
	48–59	89	22.5
**Place of Child Birth**	Health Facility	297	68.8
	Home	136	31.2
**Delivery Status**	Post term	0	0
	Term	425	98.4
	Preterm	7	1.6
**Nutritional Status (MUAC)**	**≥** 13.5cm	167	38.7
	< 13.5cm	265	61.3
**Vaccination Status**	Not vaccinated	5	1.2
	Partially Vaccinated	58	13.4
	Fully Vaccinated	369	85.4
**History of illness in two weeks**	Yes	17	3.9
	No	415	96.1
**Intestinal parasites (n = 251)**	Positive	35	13.9
	Negative	215	86.1
**Anemia status of child**	Anemic	123	28.5
	Non-anemic	309	71.5

#### Parental socio-demographic, health related and economic characteristics

Of the total respondents, almost all (98.8%) were Orthodox, whereas the remaining 0.9% and 0.3% were Protestant and Muslim religion followers, respectively. In addition, the majority (402, or 93%) of the mother respondents were married. Regarding paternal educational status, more than one-third (158; 36.6%) of the mothers cannot read and write. Moreover, 411 (95.2%) of them were housewives. Economically, nearly half of the households are classified in the lower socio-economic class. The median household size in rural and urban areas was 5, IQR (4–6) and 4, IQR (3–5) people per household, respectively. Of the total rural study participants, 134 (34.4%) did not have access to clean drinking water, but all urban respondents did. Concerning the defecation system, 376 (96.4%) of the rural and 42 (100%) of the urban respondents use traditional pit latrines and improved well-ventilated toilets, respectively. Around 72.9% of the total households suffered from household food insecurity, and almost half (49.5%) of them consumed 4–6 food groups per day (Table 2).

**Table 2:** Socio- demographic, health related and economic, characteristics of care givers in the Menz Gera Midir district Northeast Ethiopia in 2021, (N = 432).

Variable	Category	Frequency (n)	Percentage (%)
**Sex of the child care giver**	Female	420	97.2
	Male	12	2.8
**Residence**	Rural	390	90.3
	Urban	42	9.7
**Religions**	Orthodox	427	98.8
	Others	5	1.2
**Relationship of the care giver to the child**	Mother	414	95.8
	Father	12	2.8
	Others	6	1.4
**Marital Status**	Married	402	93.0
	Divorced	25	5.8
	Single	5	1.2
**Sex of the head of the household**	Male	406	93.99
	Female	26	6.01
**Occupation of Mother**	Housewife	411	95.2
	Government employed	14	3.2
	Merchant	7	1.6
**Father’s Occupation**	Farmer	365	84.5
	Governmental Employee	22	5.1
	Labor	19	4.4
	Merchant	26	6.0
**Maternal Educational status**	No Education	158	36.6
	Primary School	155	35.9
	Secondary completed	98	22.7
	Higher Education Completed	21	4.9
**Father’s Educational status**	No Education	94	21.8
	Primary School	191	44.2
	Secondary completed	125	28.9
	Higher Education complete	22	5.1
**Wealth quintile**	Higher	64	14.8
	Medium	164	38.0
	Lower	204	47.2
**Family size in the household**	< = 3	98	22.7
	4–6	275	63.7
	> = 7	59	13.6
**Type of toilet used**	Traditional Pit latrine	376	87.0
	Ventilated improved latrine	44	10.2
	Others	12	2.8
**Access to clean drinking water**	Yes	298	69
	No	134	31
**Household Food Security (HHFS)**	Food Secured	117	27.1
	Food in secured	315	72.9
**Household Dietary Diversity Score**	0–3 food groups	188	43.5
	4–6 food groups	214	49.5
	7–8 food groups	30	6.9
	> = 9 food groups	0	0.0

#### Prevalence of undernutrition

**Fig 1:**
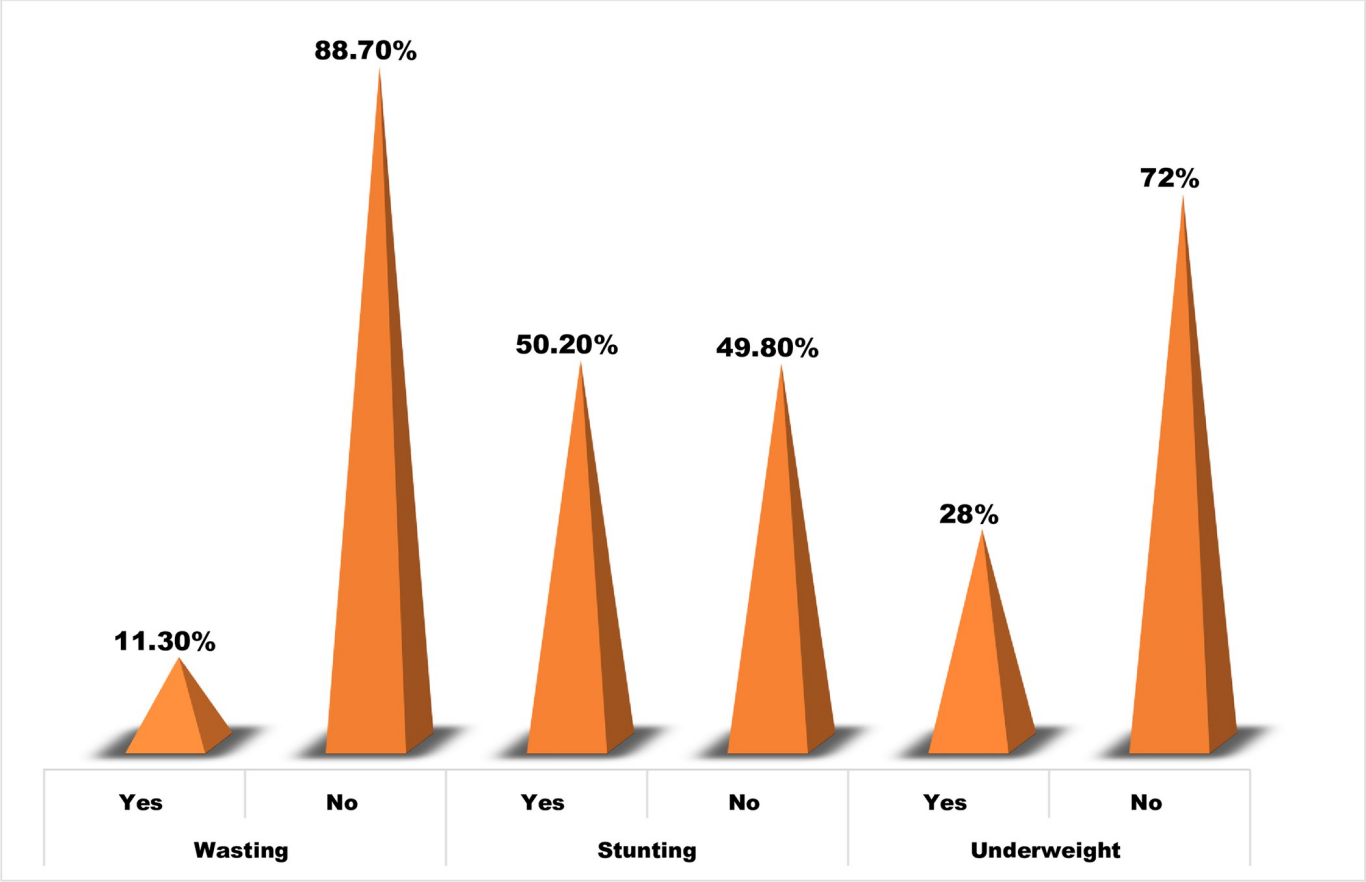
Prevalence of under-nutrition among children aged 6 to 59 months in Menz Gera Midir District, Northeast Ethiopia, in 2021 (N = 432).

From the total of 432 children, 11.3% (95% CI: 8.3–14.3%), 50.2% (95% CI: 45.5–55.0%), and 28% (95% CI: 23.8–32.3%) were wasted, stunted, and underweight, respectively **(Fig 1**).

### Factors associated with undernutrition

In bivariable logistic regression analysis, sex of the child, age of the child, anemia status of the child, the severity of food insecurity, and anemia status of the mother were significantly associated with stunting with a p-value of less than 0.2. Hence, these variables were fitted into a multivariable logistic regression model. However, only child age and child anemia remained significantly associated with stunting. The odds of stunting among children aged 12–23 and 36–47 months were 1.97 (95% CI: 1.01–3.87) and 2.05 (95% CI: 1.00–4.19) compared to children aged 6–11 months, respectively. Anemic children were 2.92 (95% CI: 1.73–4.92) times more likely to be stunted compared to non-anemic children (Table 3).

**Table 3:** Bivariable and multivariable logistic regression analysis of stunting among children aged 6 to 59 months in the Menz Gera Midir district Northeast Ethiopia in 2021, (N = 432).

Variable	Category	Stunting n (%)	COR (95% CI)	AOR (95% CI)	P value
**Sex**	Female	121 (47.6)	1	1	0.112
	Male	96 (53.9)	1.28 (0.71–1.89)	1.40 (0.92–2.13)	
**Age of the child**	6–11	33 (45.8)	1	1	
	12–23	43 (39.8)	1.54 (0.84–2.86)	**1.97 (1.01–3.87)**	0.048
	24–35	41 (56.9)	1.39 (0.74–2.63)	1.68 (0.85–3.35)	0.138
	36–47	45 (54.2)	1.56 (0.81–3.03)	**2.05 (1.00–4.19)**	0.05
	48–59	55 (56.7)	0.78 (0.43–1.43)	0.82 (0.43–1.56)	0.546
**Residence**	Rural	199 (51.0)	1.39 (0.73–2.64)	-	0.314
	Urban	18 (42.9)	1	-	
**Child anemia status**	Anemic	79 (64.2)	2.23 (1.45–3.43)	**2.92 (1.73–4.92)**	<0.001
	Non-anemic	138 (44.7)	1	1	
**Family size**	≤3	48 (49.0)	1	-	
	4–6	141 (51.3)	1.10 (0.69–1.74)	-	0.697
	≥7	28 (47.5)	0.94 (0.49–1.80)	-	0.853
**Care giver sex**	Female	211 (50.2)	1	-	0.853
	Male	6 (50.0)	0.99 (0.31–3.12)	-	
**Age of the mother**	≤30	143 (48.3)	1		0.239
	>30	74 (54.4)	1.23 (0.85–1.92)		
**Food security**	Secured	64 (54.7)	1	-	0.258
	Insecure	153 (48.6)	0.78 (0.51–1.20)	-	
**Dietary diversity score**	≤3	96 (51.1)	1	-	
	4–6	108 (50.5)	0.73 (0.34–1.59)	-	0.433
	≥7	13 (43.3)	0.98 (0.66–1.45)	-	0.905
**Anemia status of the mother**	Anemic	60 (60.6)	1.67 (1.05–2.64)	1.28 (0.76–2.13)	0.353
	Non-anemic	146 (48.0)	1	1	
**Wealth index**	Low	68 (47.2)	1	-	
	Medium	71 (49.3)	1.32 (0.83–2.10)	-	0.239
	High	78 (54.2)	1.09 (0.69–1.73)	-	0.724
**Mother occupation**	Housewife	206 (50.1)	1	-	0.840
	Others	11 (52.4)	1.10 (0.46–2.63)	-	
**Mother educational status**	No formal education	157 (50.2)	1	-	
	Certificate/ diploma	50 (51.0)	1.04 (0.66–1.63)	-	0.882
	Degree and above	10 (47.6)	0.90 (0.37–2.19)	-	0.822
**Marital status**	Married	203 (50.5)	1.17 (0.55–2.45)	-	0.686
	Single/Divorce	14 (46.7)	1	-	
**Parasite infection**	Positive	18 (43.9)	1		
	Negative	109 (51.9)	1.38 (0.70–2.71)	-	0.350
**Illness in the past 2 weeks**	Yes	9 (52.9)	1.12 (0.41–2.96)	-	0.820
	No	208 (50.1)	1	-	
**Birth order**	1–2	120 (48.2)	1	-	0.323
	≥3	97 (53.0)	1.21 (0.83–1.78)	-	

**Note**: AOR; Adjusted Odds Ratio, CI; Confidence Interval, COR; crude Odds Ratio.

On the other hand, in the bivariable logistic regression model, child anemia, family size, severity of household food insecurity, and anemia status of the mother/caregiver were predictors of wasting with a p-value of less than 0.2. However, in the multivariable logistic regression model, only child anemia was found to be significantly associated with wasting. The odds of wasting were 6.84 (95% CI: 3.16–14.82) times more likely in anemic children compared to non-anemic children (Table 4).

**Table 4:** Bivariable and multivariable logistic regression analysis of wasting among children aged 6 to 59 months in the Menz GeraMidir district Northeast Ethiopia in 2021, (N = 432).

Variable	Category	Wasting n (%)	COR (95% CI)	AOR (95% CI)	P value
**Sex**	Female	26 (10.2)	1	1	0.387
	Male	23 (12.9)	1.30 (0.72–2.36)	-	
**Age of the child**	6–11	8 (11.1)	1	-	
	12–23	16 (14.8)	1.13 (0.44–2.93)	-	0.802
	24–35	4 (5.6)	0.97 (0.36–2.67)	-	0.958
	36–47	9 (10.8)	0.47 (0.14–1.64)	-	0.236
	48–59	12 (12.4)	1.39 (0.56–3.45)	-	0.475
**Residence**	Rural	45 (11.5)	-	Fisher exact	1
	Urban	4 (9.5)	-	-	
**Child anemia status**	Anemic	33 (26.8)	6.72 (3.53–12.76)	**6.84 (3.16–14.82**	<0.001
	Non-anemic	16 (5.2)	1	1	
**Family size**	≤3	15 (15.3)	1	1	0.099
	4–6	24 (8.7)	0.53 (0.27–1.06)	0.52 (0.24–1.13)	
	≥7	10 (16.9)	1.13 (0.47–2.71)	1.60 (0.60–4.32)	0.351
**Care giver sex**	Female	48 (11.4)	-	Fisher exact	1
	Male	1 (8.3)	-	-	
**Age of the mother**	≤30	33 (11.1)	1	-	0.851
	>30	16 (11.8)	1.06 (0.56–2.01)	-	
**Food security**	Secured	11 (9.4)	1	-	0.439
	Insecure	38 (12.1)	1.32 (0.65–2.68)	-	
**Dietary diversity score**	≤3	23 (12.2)	1	-	0.901
	4–6	23 (10.7)	1.08 (0.31–3.86)	-	
	≥7	3 (10.0)	1.26 (0.35–4.47)	-	0.726
**Anemia status of the mother**	Non-anemic	24 (7.9)	1	1	0.504
	Anemic	19 (19.2)	2.77 (1.45–5.31)	1.30 (0.61–2.76)	
**Wealth index**	Poor	19 (13.2)	1	-	
	Medium	14 (9.4)	0.82 (0.41–1.67)	-	0.589
	Rich	16 (11.1)	0.71 (0.34–1.47)	-	0.357
**Mother occupation**	Housewife	45 (10.9)	-	Fisher exact	0.280
	Others	4 (19.0)	-	-	
**Mother educational status**	No formal education	35 (11.2)	-	Fisher exact	0.884
	Certificate/ diploma	11 (11.2)	-	-	
	Degree and above	3 (14.3)	-	-	
**Marital status**	Married	47 (11.7)	-	Fisher exact	0.558
	Single/Divorce	2 (6.7)	-	-	
**Parasite infection**	Positive	2 (4.9)	-	Fisher exact	0.155
	Negative	21 (10.0)	-	-	
**Illness in the past 2 weeks**	Yes	2 (11.8)	-	Fisher exact	0.595
	No	47 (11.3)	-	-	
**Birth order**	1–2		1	-	0.590
	≥3		0.85 (0.46–1.56)	-	

**Note:** AOR; Adjusted Odds Ratio, CI; Confidence Interval, COR; crude Odds Ratio.

In bivariable logistic regression analysis of factors associated with underweight, age of the child, residence, child anemia status, family size, and age of mother/caregiver, anemia status of the mother, wealth index, and parasite infection were predictors of underweight at p-value less than 0.2. However, in multivariable logistic regression, controlling for the cofounder, all of the predictors were not statistically associated with underweight (Table 5).

**Table 5:** Bi-variable and multi-variable logistic regression of underweight among children aged 6 to 59 months in the Menz Gera Midir district Northeast Ethiopia in 2021, (N = 432).

Variable	Category	Underweight n (%)	COR (95% CI)	AOR (95% CI)	P value
**Sex**	Female	74 (29.1)	1	-	0.534
	Male	47 (26.4)	0.87 (0.57–1.34)	-	
**Age of the child**	6–11	24 (33.3)	1	1	
	12–23	31 (28.7)	0.81 (0.42–1.53)	0.77 (0.39–1.54)	0.461
	24–35	19 (26.4)	0.72 (0.35–1.47)	0.87 (0.40–190)	0.718
	36–47	26 (31.3)	0.91 (0. 47–1.79)	1.03 (0.49–2.16)	0.0.939
	48–59	21 (21.6)	0.55 (0.28–1.10)	0.75 (0.35–1.58)	0.455
**Residence**	Rural	114 (29.2)	1	1	0.245
	Urban	7 (16.7)	0.48 (0.21–1.12)	0.59 (0.24–1.44)	
**Child anemia**	Non-anemic	72 (23.3)	1	1	0.062
	Anemic	49(39.8)	2.18 (1.39–3.41)	1.68 (0.98–2.88)	
**Family size**	≤3	32 (32.7)	1	1	0.207
	4–6	68 (24.7)	0.88 (0.45–1.73)	0.69 (0.39–1.23)	
	≥7	21 (35.6)	0.59 (0.33–1.08)	1.50 (0.67–3.32)	0.323
**Care giver sex**	Female	119 (28.3)	-	Fisher exact	0.523
	Male	2 (16.7)	-	-	
**Age of the mother**	≤30	92 (31.1)	1	1	0.099
	>30	29 (21.3)	0.60 (0.37–0.97)	0.62 (0.35–1.10)	
**Food security**	Secured	31 (26.5)	1	-	0.669
	Insecure	90 (28.6)	1.11 (0.60–1.79)	-	
**Dietary diversity score**	≤3	51(27.1)	1	-	
	4–6	59 (27.6)	0.66 (0.30–1.46)	-	0.305
	≥7	11(36.7)	0.64 (0.29–1.44)	-	0.285
**Anemia status of the mother**	Anemic	33 (33.3)	1.45(0.88–2.37)	1.20 (0.69–2.09)	0.520
	Non-anemic	78 (25.7)	1	1	
**Wealth index**	Poor	48 (33.3)	1.50 (0.90–2.50)	1.08 (0.58–2.03)	0.808
	Medium	37 (25.7)	1.04 (0.61–1.76)	0.90 (0.50–1.65)	0.744
	Rich	36 (25.0)	1	1	
**Mother occupation**	Housewife	115 (28.0)	1	-	0.953
	Others	6 (28.6)	1.03 (0.39–2.72)	-	
**Mother educational status**	No formal education	93 (29.7)	1.06 (0.40–2.81)	-	0.912
	Certificate/ diploma	22 (22.4)	0.72 (0.25–2.09)	-	0.549
	Degree/above	6 (28.6)	1	-	
**Marital status**	Married	113 (28.1)	1	-	
	Single/Divorce	8 (26.7)	1.08 (0.47–2.49)	-	0.856
**Parasite infection**	Yes	9 (22.0)	1	1	0.903
	No	52 (24.8)	1.70 (0.52–2.61)	1.06 (0.45–2.50)	
**Illness in the past 2 weeks**	Yes	2 (11.8)		Fisher exact	0.101
	No	119 (28.7)	-	-	
**Birth order**	1–2	74 (29.7)	1	-	0.356
	≥3	47 (25.7)	0.82 (0.53–1.26)	-	

Note: AOR; Adjusted Odds Ratio, CI; Confidence Interval, COR; crude Odds Ratio.

## Discussion

To prevent the health complications of under five children attributable to undernutrition, it is important to assess the prevalence of undernutrition and factors associated with its prevalence. Therefore, the main objective of this study was to determine the prevalence of undernutrition and assess associated factors among under-five children. The finding revealed that the prevalence of stunting, wasting, and underweight was 50.2% (95% CI: 45.5–55.0%), 11.3% (95% CI: 8.3–14.3%), and 28% (95% CI: 23.8–32.3%), respectively. The findings were high, which revealed that undernutrition is still a public health problem in Ethiopia, especially in the Amhara region. According to the 2016 EDHS report, undernutrition was a public health problem in the Amhara region [33].

The prevalence of stunting (50.2%) among children aged 6–59 months in this study was comparable with the results of other similar studies conducted in Hawassa, southern Ethiopia (53.1%) [34], Bule Hora district, South Ethiopia (47.6%) and Ilu Abba Bora Zone, Southwest Ethiopia (50.8%) [35]. It was also in line with the findings in India; Madhya Pradesh, Jabalpur (51.6%) [36] and Rajasthan, Jodhpur districts (53%) [37]. However, the finding was higher than other studies conducted in east Gojjam, northwest Ethiopia (44.7%) [38], slum areas of Gondar city, northwest Ethiopia (42.3%) [39], Somali region, eastern Ethiopia (27.4%) [40], Jimma, Southwest Ethiopia (21.8%), [2] orphanage centers, and Addis Ababa, Ethiopia (34.8%) [41]. Moreover, it was higher than other east African countries, including Tanzania; Kilosa District (41.0%) [42] and Rwanda; Ngoma District (33.7%) [43]. It was 6–13 times higher than the studies conducted in China [44–46]. It was also higher than the study conducted in central India (34.8%) [47] and the city of Maharashtra, India (40.5%) [48] and Roma, Italy (11.7%) [49]. On the other hand, the current finding was lower than studies done in Dabat District, northwest Ethiopia (64.5%) [50], and East Belesa District, northwest Ethiopia (57.7%) [51]. The possible reasons for this variation might be socio-demographic and socio-economic differences and the difference in geographic settings. Socio-demographic and socio-economic status have an effect on the nutritional status of children [47,48,52]. Geographic location also affects the cultivation of food crops, which in turn affects access and availability of food for household consumption. The impact of geographical location could be because of environmental variability in the occupation practiced, which could influence food security and consequently affect child nutrition, growth and development. Geographic locations are also favorable for climatic conditions, which affect food production [53].

Previous studies showed that the prevalence of stunting was significantly associated with family size, wealth index, child age, food security, source of drinking water, and dietary diversity [35,54–57]. However, in the current study, only child age and child anemia status were significantly associated with stunting. Children aged 12–23 and 36–47 months were about 2 times more likely to be stunted compared to children aged 6–11 months (AOR; 1.97 (95% CI: 1.01–3.87) and 2.05 (95% CI: 1.00–4.19, respectively). The finding was in line with a study done in southern Ethiopia [54]. East African Districts (Rwanda’s Gicumbi District, Uganda’s Kitgum District, and Tanzania’s Kilindi District) [53], India [48] and China [45]. This might be because of a high prevalence of childhood stunting, which reflects undernutrition, starts in the first years of life (6–24 months) [58] and becomes worse at different phases of growth and results in short adult stature [59]. The older children undergo rapid growth, which creates a high demand for energy and other nutrients. Moreover, for older children, the only source of nutrients was complimentary foods, while the younger children benefited from both breast milk and complimentary foods. These factors might compromise the child’s nutritional status at this age [42]. An increased interaction of the older child with its immediate environment might also be the other reason. Environmental interaction leads to an increased risk of infections and exposure to childhood diseases either through drinking of unimproved water sources, consumption of contaminated foods, poor hygiene, or poor environmental sanitation [60].

The odds of stunting among anemic children were 2.92 times more likely compared to their counterparts. This finding was consistent with studies conducted in Ethiopia [33,61]. Undernourished children are more likely to suffer from inadequate bioavailability of micronutrients such as iron, vitamin B12, and folate, which are important for the formation of blood cells. Undernourished children are unable to produce as many blood cells as needed, leading to the development of nutritional deficiency anemia, which is common, particularly in developing countries [62].

In the current study, the prevalence of wasting (11.3%) was similar to studies conducted in Damot Gale district, south Ethiopia (9%) [63], Bule Hora district, South Ethiopia (13.4%) [4], and the east Gojjam zone, Northwest Ethiopia (10–11.3%) [38]. However, it was lower than other studies conducted in the Somali region, Ethiopia (22.7%) [33], Hawassa, Southern Ethiopia (28.2%) [34], East Belesa District, Northwest Ethiopia (16%) [51], North Sudan (21%) [64], Australia (15%) [65] and Maharashtra, India (16%-17.1%) [48,66]. On the other hand, the current finding was higher than studies conducted in Gondar Town, Northwest Ethiopia (6.8%) [67], slum areas of Gondar City, Northwest Ethiopia (7.3%) [39], orphanage centers, Addis Ababa, Ethiopia (4.4%) [41], Ngoma District, Rwanda (3.6%) [68], South Sudan (2.3%) [69], Italy (2.9%) [49], and China (2–4%) [44–46]. This might be due to the difference in the sample size, study setting, socio-demographic characteristics, socio-economic status, geographic location, and study period.

According to the previous studies, a number of determinants of wasting were identified. These include exclusive breast feeding, acute diarrhea [66], family size [46] presence of a fever in the previous 2 weeks [39] and illness in the last two weeks [41]. In the current study, only child anemia was found to be significantly associated with wasting. The odds of wasting were 6.84 times more likely in anemic children compared to their counterparts. The finding was in agreement with studies done in Menz Gera Midir district, Eastern Amhara, Ethiopia [70], and rural areas of Shaanxi, northwestern China [71]. Anemia and undernutrition often have common causes. Undernutrition and anemia have an interplaying association. Childhood anemia might occur as a result of a macro-nutrient deficiency or it precipitates the occurrence of under-nutrition owing to the poor synthesis of macronutrients. In other words, undernourished children more often suffer from inadequate availability of micronutrients such as iron, B12, and folate, which are important for erythropoiesis. Therefore, those children who are undernourished will have impaired production of adequate red blood cells as much as required. Consequently, this leads to the development of nutritional deficiency anemia [62,70,72].

This study also showed that the prevalence of underweight was 28%. This finding was consistent with other findings reported in the Damot-Galle district, south Ethiopia (27.6%) [63], Hawassa, Southern Ethiopia (28.2%) [34], Bule Hora district, South Ethiopia (29.2%) [4], and Kerala, India (28.3%) [73]. However, it was higher than the studies conducted in East Gojjam zone, Northwest Ethiopia (15.3%) [38], East Belesa District, northwest Ethiopia (16%) [51], Takusa district, Northwest Ethiopia (19.5%) [74], Jimma town, southwest Ethiopia (15.2%) [2], orphanage centers, Addis Ababa, Ethiopia (12.3%) [41], South Sudan (4.8%) [75], Ngoma District, Rwanda (6.6%) [68] and Kilosa District, Tanzania (11.5%) [42]. In contradiction, the result was lower than the studies conducted in urban slums and rural areas of Maharashtra, India (35.4%) and the urban slums of Pune (34.3%) [76]. Differences in socioeconomic, cultural, and child feeding patterns, seasonal variance in study time, and age group of the study population, study setting, and genetic factors could all contribute to the disparity.

According to this study, undernutrition is a community health problem in the Menz-Gera Midir district. Therefore, policies which are currently applied in the study area should be considered that children’s, specifically those who are aged 6 to 59 months, are highly affected by undernutrition, and they also need an advanced policy to eliminate undernutrition in the study area.

This study has its strength, which uses a concentration technique that helps to identify lower parasitic load. However, the major limitation of this study is that the micronutrient status of children was not measured (except anemia) and its real association with undernutrition was not known. Moreover, subclinical infections other than intestinal parasites were not assessed, which could limit the generalizability of the findings. Besides, as the study is cross-sectional, no causal link can be inferred.

## Conclusion

In this study, undernutrition was a highly prevalent health problem among 6–59 month old children in the Menz Gera Midir District. This shows that early intervention, including community-based nutrition education programs and health programs, is vital to reduce childhood stunting, wasting, and being underweight, especially in the first 2 years of life. Childhood anemia and child age were linked to stunting. On the other hand, only anemia was a risk factor for wasting. As a result, community-based intervention and implementation should be strengthened in order to reduce the effect of anemia and the high burden of undernutrition among children under the age of five.

## Abbreviations

AOR: Adjusted Odds Ratio
COR: crude odds ratio
EDHS: Ethiopian demographic health survey
HAZ: Height for age
Hgb: Hemoglobin
MUAC: Mid-upper Arm Circumferences
WAZ: weight-for-age
WHZ: Weight for height
WHO: World Health Organization
